# Changes to telehealth practices in primary care in New Brunswick (Canada): A comparative study pre and during the COVID-19 pandemic

**DOI:** 10.1371/journal.pone.0258839

**Published:** 2021-11-23

**Authors:** Claire Johnson, Jérémie B. Dupuis, Pierre Goguen, Gabrielle Grenier

**Affiliations:** 1 School of Public Policy Studies, Université de Moncton, Moncton, New Brunswick, Canada; 2 Faculty of Education, Université de Moncton, Moncton, New Brunswick, Canada; 3 Champlain Library, Université de Moncton, Moncton, New Brunswick, Canada; Ohio State University Wexner Medical Center Department of Surgery, UNITED STATES

## Abstract

**Background:**

During the COVID-19 pandemic, telehealth technologies were used in the primary health care setting in New Brunswick as a means to continue providing care to patients while following public health guidelines. This study aimed to measure these changes and examine if they improved timely access to primary care. A secondary goal was to identify which telehealth technologies were deemed sustainable by primary care providers.

**Methods:**

This was a comparative study on the use of telehealth technology before and during the COVID-19 pandemic. Between April 2020 and November 2020, 114 active primary care providers (family physicians or nurse practitioners) responded to the online survey.

**Results:**

The findings illustrated an increase in the use of telehealth technologies. The use of phone consultations increased by 122%, from 43.9% pre-pandemic to 97.6% during the pandemic (*p* < 0.001). The use of virtual consultation (19.3% pre-pandemic vs. 41.2% during the pandemic, *p* < 0.001), emails and texts also increased during the pandemic. Whereas the more structural organizational tools (electronic medical charts and reservation systems) remained stable. However, those changes did not coincide with a significant improvement to timely access to care during the pandemic. Many participants (40.1%) wanted to keep phone consultations, and 21.9% of participants wanted to keep virtual consultations as part of their long-term practice.

**Interpretation:**

The observed increase in the use of telehealth technologies may be sustainable, but it has not significantly improved timely access to primary care in New Brunswick.

## Introduction

During the COVID-19 pandemic, many changes were made to the primary health care setting in New Brunswick in order to continue providing care to patients while following public health guidelines [1]. Primary health care is known to be the cornerstone and the first point of contact in most healthcare systems in developed countries [2, 3]. Therefore, it was crucial to continue operations even in the context of a global pandemic [4] since primary care is the best method to prevent hospitalizations and visits to the emergency department. As social distancing was a key measure in preventing the spread of COVID-19 and other communicable diseases by reducing person-to-person contact [5], many primary care providers were forced to increase use of virtual health or telehealth [6] as a means to continue providing care [7, 8]. There have been many reports of an increase in the use of virtual care to provide primary care to New Brunswickers [9, 10]. However, the degree to which telehealth (phone consultation, virtual consultation, or text messaging) has increased during the COVID-19 pandemic is not known. In addition, many experts have expressed their enthusiasm for virtual care, stating it should be implemented proactively (as opposed to reactively as seen during this pandemic) as a way to efficiently manage everyday or emergency challenges in primary care [5]. In addition, claims that telehealth technologies may improve access to care are often made, but those claims often lack robust evidence to support them [11].

In a survey conducted in the general population of New Brunswick, timely access to primary care has been identified as a barrier to quality of care, as 44% of respondents reported not being able to access their primary care provider within five days or less [12]. There is convincing evidence that technology usage could be a tool to help improve timely access to primary care [6, 13]. The use of technology (such as those found in telehealth and telemedicine) and timely access to primary care are important indicators for quality of care used by experts in the field of healthcare management [14, 15] and by patients themselves [3].

This study aims to assess the changes in the use of telehealth technology in primary care and other organizational practices during the COVID-19 pandemic. The changes in telehealth technology use are the independent variables, and timely access to primary care is the outcome variable [13]. It was hypothesized that there was an increase in technology usage (phone consultations, virtual consultation, and text messaging, for example) during the pandemic and that the uptake in technology usage are associated with an improvement in timely access to primary care in New Brunswick. Timely access to primary care is essential, and especially in New Brunswick, where it is believed to be the underlying reason that many people to use the emergency department for non-urgent issues [12].

As a secondary goal, we verified satisfaction among primary care providers regarding changes to telehealth technology usage and other practices during the COVID-19 pandemic. This was to identify which changes to their practice were deemed helpful and could be sustainable post-pandemic. It was hypothesized that practices deemed helpful by providers may improve timely access to care and could be integrated as part of primary care post-pandemic. Results from this secondary goal could help policy-makers in New Brunswick assess which services to continue to fund through public insurance coverage (“Medicare”) [16, 17] once the COVID-19 pandemic is over. These policy decisions are key because they can represent economic barriers to telehealth sustainability [6].

### Theoretical framework

**Fig 1:**
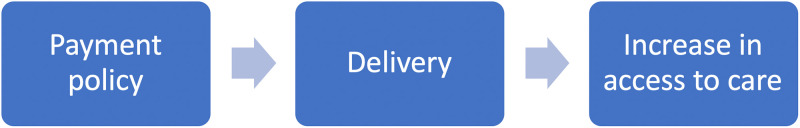
Telehealth research framework.

Edmunds et al., 2017 proposed a telehealth research framework that highlights the relationship between payment policy, telehealth delivery and increased access to care [13]. Although their framework contains other indicators, a section illustrates how payment policy decisions influence delivery which ultimately influences increased access to care. Fig 1 below was taken from the Edmunds et al. framework for this study [13]:

## Methods

### Study design and study population

This is a comparative study on the use of technology before and during the COVID-19 pandemic using an online survey between April and November 2020. Participants for this study are active primary care providers, such as family physicians or nurse practitioners, who provide first-contact services and were working in New Brunswick. We decided to focus on family physicians and nurse practitioners because they are chiefly responsible for delivering primary care in New Brunswick and Canada [18]. For feasibility reasons, 500 primary care providers were systematically sampled from a list of 2000 primary care providers provided by the New Brunswick Department of Health. The sample strategy was to contact every fourth provider from the list. Each potential participant was contacted by phone at their office to see if they wanted to participate in the study. Of the 500 potential participants contacted, 114 participants volunteered and responded to our online survey, resulting in a 22.8% response rate. This convenience sample was deemed representative of the primary care provider population in New Brunswick since all of the province’s seven health zones were represented in the sample (see Table 1). On a smaller scale, 26 of the province’s 33 communities were also represented. Non-active primary care providers, physicians, and nurses working outside primary care (emergency care, long-term care or specialized medical care) were excluded from this study.

**Table 1:** Participants’ geographical region and affiliated health network.

Participant	Frequency	Proportion
	*n*	%
Health Zones		
Zone 1 (Moncton/South-East Area)	32	29%
Zone 2 (Fundy Shore/Saint John Area)	16	15%
Zone 3 (Fredericton/River Valley Area)	15	14%
Zone 4 (Madawaska/North-West Area)	19	17%
Zone 5 (Restigouche Area)	8	7%
Zone 6 (Bathurst and Acadian Peninsula Area)	17	16%
Zone 7 (Miramichi Area)	3	3%
Missing	4	
Health Network		
Vitalité-Francophone	61	54.5%
Horizon- Anglophone	51	45.5%
Missing	2	

### Survey

A survey with dichotomous questions regarding the use of telehealth technologies and open-ended questions on the challenges and the perceived potential for sustainability of new practices acquired during the COVID-19 pandemic was used to collect data. The survey was developed as a result of a literature review on telehealth, telemedicine, technology usage, and timely access to primary care. To assess the use of telehealth and other technologies and their influence on timely access to primary care, participants were asked to answer “yes” or “no” as to whether they used email [8, 19], electronic medical records [14, 20–22], a digital reservation system [14, 23, 24], texts, voicemail, videoconference [19, 20], telephone consultation [19, 20], telework or if they worked remotely from home. Then, to assess the change during the pandemic for each indicator, participants were asked: “Did you use (each technology) prior to the COVID-19 pandemic. Answer: yes or no” and “Did you use (each technology) during the COVID-19 pandemic. Answer: yes or no”.

For timely access, participants were asked the number of days patients had to wait to see them for an urgent request [12, 14]. Participants were also asked how many patients were seen daily, what were their hours of operation, if they offered care outside regular office hours, and then asked if this was for the pre-pandemic period or during the pandemic [14]. Lastly, participants were asked about the total number of patients under their care [25]. To assess which new practices should be maintained once the COVID-19 pandemic is over, the following open-ended question was asked: “Which new practice would you like to keep once the COVID-19 pandemic is over?”. To lessen the impact of social desirability bias, the questions were neutral and factual to avoid attaching value to the possible responses.

### Data collection and analysis

Potential participants were contacted at their office by telephone to solicit participation. Once they expressed a willingness to participate in the study, they were sent an email with a consent form and a link to the online survey. When requested, they were sent the consent form and questionnaire by fax. We used SPSS by IBM to perform descriptive and chi-square analysis to compare the use of telehealth tools before and during the COVID-19 pandemic. All significance levels were set at *p* < 0.05 for every statistical analysis.

To analyze the qualitative part of the survey, responses to the open-ended questions were transcribed into NVivo for coding by theme. Common themes were identified to draw connections between ideas to establish thematic saturation. The themes were established based on responses most often reported by participants. During the data collection and analyses, meetings and discussions between the research assistants and the principal researcher (professor) occurred regularly to analyze the thematic content and to organize grouping categories of data and information. The analysis was done by three different people (2 research assistants and one professor), and the results were similar enough to validate the findings (within 5% variation).

### Ethics approval

This study was approved by the research ethics board at the Vitalité Health Network and the Université de Moncton (file #1920–051).

## Results

Table 1 presents the participants’ geographical region and affiliated health network. In New Brunswick, the province is divided into seven health zones and each zone is affiliated with one of the two health networks (Vitalité and Horizon).

Table 2 illustrates an increase in the use of certain telehealth technologies during the COVID-19 pandemic, whereas others remained stable. The most significant increase was for the use of phone consultations; they jumped from 43.9% pre-pandemic to 97.6% during the pandemic (*p* < 0.001), indicating a 122% increase in use. Following similar patterns, the use of virtual (or video chat) consultation and telework (working from home or working remotely) jumped from 19.3% pre-pandemic to 41.2% during the pandemic (*p* < 0.001) and from 15.9% to 41.2% (*p <* 0.001) respectively. The most common “other technologies” reported was the use of applications (apps). Emails and text usage also increased during the pandemic, whereas the more structural organizational tools (reservation system and electronic medical charts) remained stable.

**Table 2:** Telehealth technology usage pre-pandemic and during the COVID-19 pandemic (*n* = 114).

Technology	Pre-pandemic	Pandemic	*p* value of chi-square
	Usage	Usage	
Consultation telehealth technologies:			
Email	61.3%	75.6%	0.036*
Texts	8.3%	18.8%	0.029*
Voicemail	54.1%	54.1%	0.999
Virtual consultations	19.3%	41.2%	< 0.001*
Telephone Consultations	43.9%	97.6%	< 0.001*
Other technologies	1.8%	10.6%	0.009*
Structural organizational tools:			
Electronic medical records	71.6%	68.6%	0.654
Reservation system	12.8%	11.6%	0.797
Working remotely	15.9%	41.2%	< 0.001*

Note.

* Indicates a significance level of *p* < 0.05.

Table 3 shows a higher proportion of patients (92.3%) had timely access to primary care during the COVID-19 pandemic compared to the proportion of patients who had the same access before the pandemic (79.2%). However, the difference was not significant (*p =* 0.070).

**Table 3:** Timely access to primary care indicators pre-pandemic and during the COVID-19 pandemic (*n* = 114).

	Indicator	Pre-pandemic	Pandemic	p-value of chi-square
Wait time for urgent request	5 days or less	79.2%	92.3%	0.070
After hours access	Yes	24.3%	16.7	0.194

Note. Significance levels were set at *p* < 0.05 for all statistical analysis.

The results in Table 4 suggest that demand for primary care may have been lower during the COVID-19 pandemic since a significant reduction in the average number of patients seen per day was reported (25 patients pre-pandemic vs. 18 patients during the pandemic, *p* < 0.001). Overall, although the results form Table 3 suggest that timely access during the pandemic may have improved slightly, the improvement may be due to lower demand. A reduction in demand for primary care services was also observed, as illustrated by a significant drop in the average number of patients seen per day. In support of this observation, other timely access indicators remained stable (after-hour access, office hours, number of patients in providers’ care), suggesting a reduction in demand for primary care during the pandemic.

**Table 4:** Timely access to primary care indicators pre-pandemic and during the COVID-19 pandemic (*n* = 114).

		Average	*p* value t-test	Standard error difference
Patients seen per day	Pre-pandemic	24.6	< 0.001	1.57
	Pandemic	18.2		
Office hours	Pre-pandemic	31.9	0.593	1.48
	Pandemic	31.1		
Number of patients in providers’ care	Pre-pandemic	1269.9	0.545	117.63
	Pandemic	1341.2		

Note. Significance levels were set at *p* < 0.05 for all statistical analysis.

The results in Table 5 illustrate that most participants wish to see telehealth technologies adopted during the COVID-19 pandemic remain a part of their practice long-term. Telephone consultations, the practice that increased the most during the COVID-19 pandemic, was also the tool that most participants (68.9%) wished to keep.

**Table 5:** Physicians’ perception on the sustainable use of telehealth technology in their primary care practice post-pandemic (*n* = 114).

Telehealth technology	Proportion of participants
Telephone consultations	68.9%
Virtual consultations	23.0%
Email	4.9%
Other (Texts and open access appointment system)	3.2%

## Interpretation

The main finding of this study was an increase in the use of telehealth technology in primary care in New Brunswick during the COVID-19 pandemic. We observed a significant increase in the use of phone consultations, virtual consultations, emails, texts, and physicians working remotely. This quick uptake of virtual care during the pandemic does not appear to have significantly improved timely access to care in New Brunswick for the time being. These findings are similar to those found worldwide, where an acceleration in the use of telemedicine during the COVID-19 pandemic was reported to have helped reduce person-to-person contact while providing medical care [8, 26]. Most experts seem to agree that the use of telemedicine was helpful in reducing the spread of the virus during the COVID-19 pandemic [8]. Data from our open-ended questions illustrated that this is also the general belief among the participants. In addition, this study found that most primary care providers (91.9%) wish to continue with telehealth technology as part of their primary care practice, but to a lesser extent than during the COVID-19 pandemic. Moreover, most participants wish to see some form of telehealth remain post-pandemic but agree that a new balance between telehealth and face-to-face visits must be struck. Overall, participants felt telehealth was “overused” (albeit necessary to reduce the risk of spreading the virus) during the pandemic and wish to see less telehealth technology maintained long-term. Overall, most challenges reported by participants were associated with the COVID-19 pandemic and with public health measures rather than with telehealth technologies themselves. For example, many participants found it difficult to wear masks, maintain physical distancing or to pose a diagnosis without seeing or assessing the patient in person, especially patients they would have otherwise seen in person. They also reported finding it difficult to incorporate new telehealth practices with very little time to prepare. This may explain why phone consultations were preferred compared to other telehealth technology tools that are typically more involved [11]. A reduction in missed appointments, often referred to as “no-shows”, was a common benefit reported by participants with the increase in use of telehealth technologies.

Based on the 2019 results from the Commonwealth Fund’s International Health Policy Survey of Primary Care Physicians, the proportion of New Brunswick physicians who used more modern electronic methods, like texting, voicemail, or virtual consultations, was 12%, which is below the Canadian average of 23%, and far below the average for physicians from the Commonwealth Funds of 65% [14]. This tendency may explain why, during the uptake of telehealth observed during the COVID-19 pandemic, New Brunswick primary care providers preferred using telephone consultations, a less foreign technology to them. In fact, participants reported an increase from 43.9% to 97.6% in usage of telephone consultations (122% increase, *p* = 0.001). Other types of more modern telehealth tools (virtual consultations, texts, and emails) increased too but less than telephone consultations. A study from the United States found that many providers preferred phone consultations because they are inexpensive, easy, quick and equitable, since most people have access to a telephone [27]. That same study also found that patients over 50 years of age prefer phone calls over other telehealth tools [27], offering a possible explanation for the popularity observed in this study since the New Brunswick population has a high proportion (44.8%) of citizens over the age of 50 [28]. Telephone consultations were helpful to overcome typical barriers to care experienced by vulnerable populations with regards to childcare or work responsibilities [27]. Lastly, telephone consultations do not increase the workload for physicians as much as other telehealth technologies. Telephone consultations are estimated to only increase workload by 3% compared to around 25–30% for the use of online communication tools or virtual consultations (video-conferencing) [11]. This could also explain why primary care providers preferred phone consultations during the COVID-19 pandemic, given the short timeframe to plan and organize the use of telehealth technologies.

The proportion of participants who used virtual consultations increased from 19.3% to 41.2% (a 113.5% increase, *p* = 0.001) during the COVID-19 pandemic. A study in Ontario, Canada, reported the proportions of Ontarians who received a virtual visit increased from 1.3% in 2019 (pre-pandemic) to 29.2% in 2020 (during the pandemic) [29]. The increase in the Ontario study was higher than the findings in this study, mostly because usage pre-pandemic was lower (1.3% in Ontario vs. 19.3% in New Brunswick). Another potential discrepancy between our findings was with the measured outcomes. Our study measured telehealth technology increase from the providers’ perspective, whereas the Ontario study measured the proportion of patients who received a virtual medical visit. In Ontario, similarly to New Brunswick, payment policies were changed to include phone consultation and virtual care reimbursement for physicians. In both provinces virtual consultations were only covered when done following strict regulations and phone consultation were not covered prior to the COVID-19 pandemic. In both provinces those restrictions were lifted (or loosened) in March 2020 [29]. Multiple studies have documented an increased use of telehealth during the COVID-19 pandemic in Canada, the United States, and worldwide. However, few studies measured the increase in use [8, 19, 20, 26, 30]. Even with the increase in the usage of telehealth technologies during the COVID-19 pandemic, New Brunswick remained a low telehealth user compared to other countries from the Commonwealth fund [14]. Among physicians in the Commonwealth fund, there were countries where the vast majority of physicians used telehealth technologies [14]. In Sweden, for example, 95% of primary care physicians used telehealth technologies to communicate with their patients, 81% of physicians in Switzerland, and 79% of the physicians in the United States provided similar services [14]. The average for all Commonwealth fund countries where their primary care physicians used telehealth technologies was 63%, whereas the proportion was only 23% for Canadian primary care physicians [14]. Within Canada, New Brunswick was among the provinces with the smallest proportion of primary care physicians who used telehealth technologies, with only 12% of physicians who reported using them [14]. Participants from this study reported economic barriers [6] to technology usage in New Brunswick. Prior to the COVID-19 pandemic, phone consultations were not covered by the New Brunswick public medical insurance (“Medicare”). That means physicians could not bill the province when they provided phone consultations. Since the COVID-19 pandemic, primary care providers can charge the provincial government the same fees for in-person consultations as for phone consultations or virtual consultations. However, it remains to be seen what payment policy will be implemented post-pandemic. Since 2016, primary care and specialised physicians in neighbouring Nova Scotia were allowed to bill the Nova Scotia provincial Health Department for phone consultations [31]. However, the participants of this study confirmed that physicians in New Brunswick were only granted this same privilege during the COVID-19 pandemic. As for other telehealth technologies, virtual consultations were covered as part of the publicly funded insurance plan but only following strict regulations, for example, consultations had to be done from approved hospital sites only [16]. Whereas, during the COVID-19 pandemic, there was loosening of this restriction, and primary care providers could provide care remotely. Thus, illustrating how payment policy can influence telehealth tools, since 41.2% of participants reported working remotely during the COVID-19 pandemic compared to 15.9% pre-pandemic (statistically significant increase, *p* < 0.001). Working remotely or teleworking has increased in many areas in New Brunswick (and elsewhere in Canada) since the COVID-19 pandemic [32]. In the case of New Brunswick, payment policy changes reported during the COVID-19 appear to be influential in increasing the use of telehealth, as illustrated in the findings and the research framework used in this study (Fig 1) [13].

However, unlike the research framework used in this study (Fig 1), the findings did not illustrate a statistically significant improvement in timely access to primary care during the COVID-19 pandemic. Although the findings illustrated a statistically significant increase in the use telehealth technologies, it did not translate into a significant increase in timely access to care for the time being. Those findings could be explained in part by a decrease in requests for consultation with primary care physicians during the COVID-19 pandemic, as the results of this study suggested. During the COVID-19 pandemic, a higher proportion of patients (92.3% during the pandemic vs. 79.2% pre-pandemic, *p* = 0.07) had access to their primary care physician within five days. Therefore, suggesting an increase in efficiency during this period. However, the number of patients seen per day was significantly lower during the pandemic (18 patients per day during the pandemic vs. 25 patients per day pre-pandemic, *p* < 0.001), which suggested an overall decrease in requests for primary care. When assessed together, the higher proportion (not statistically significant) of patients seen within five days and the lower number of patients seen per day (statistically significant), the findings illustrated a possible decrease in demand instead of an improvement in timely access to primary care during the COVID-19 pandemic. These findings align with other studies that have found a reduction in demand for primary care during the COVID-19 pandemic [7, 33, 34]. Specifically, those studies found that between 2019 (pre-pandemic) and 2020 (pandemic), there was a shift towards telehealth, and the demand for consultations for COVID-19 related symptoms increased. In contrast, demand for consultations for other acute diseases and chronic disease decreased and demands for consultations for mental health remained stable [34]. Considering the reported increase in practices known to improve access to care (increased use of telehealth technologies, for example), an improvement in timely access to care in the future may be possible. That remains to be verified in future studies.

### Limitations

This study has limitations to consider. For instance, the observational nature of the study precludes inferences on causality. Furthermore, the study used a convenience sample, the data gathered was self-reported by primary care providers and therefore only presented their perspective, which was subject to social desirability bias, and failed to include patients’ perspectives.

## Conclusion

This study illustrated an increase in the use of telehealth technologies in primary care in New Brunswick during the COVID-19 pandemic, especially use of telephone consultations, followed by virtual consultations and other technologies (email and texts). Many primary care providers confirmed wanting to keep many of these initiatives post-pandemic. Additionally, participants reported a preference for keeping the two technologies most used during the COVID-19 pandemic (telephone and virtual consultations) as part of their practice long term. However, this observed uptake in telehealth technologies did not coincide with a significant improvement in timely access to primary care in New Brunswick. The findings that suggested an improvement were possibly related to the decrease in demand for primary care during the COVID-19 pandemic. This is important information for policy makers in New Brunswick who will be called upon to decide which telehealth technologies will be covered post-pandemic. Further research is needed to assess patients’ perspectives on telehealth technology usage in New Brunswick. Furthermore, more research is required to confirm if the observed increase in telehealth technologies could improve timely access to primary care post-pandemic once the demand for primary care goes back to normal.

## Supporting information

S1 Fig Telehealth framework. Republished from Edmunds et al. [13] under a CC BY license, with permission from the author, original copyright (2017).
(TIFF)

S1 Questionnaire (DOCX)

S1 Dataset (SAV)

S2 Dataset (SAV)

S1 File Table syntax Tables 1 and 2 (Database 1). (SPS)

S2 File Table syntax Tables 2, 3 and 4 (Database 2). (SPS)
