# Supplementary figures and images for: Changes to telehealth practices in primary care in New Brunswick (Canada): A comparative study pre and during the COVID-19 pandemic
Source: PLoS One. 2021 Nov 23;16(11):e0258839. doi: 10.1371/journal.pone.0258839 (PMC8610241; doi:10.1371/journal.pone.0258839)

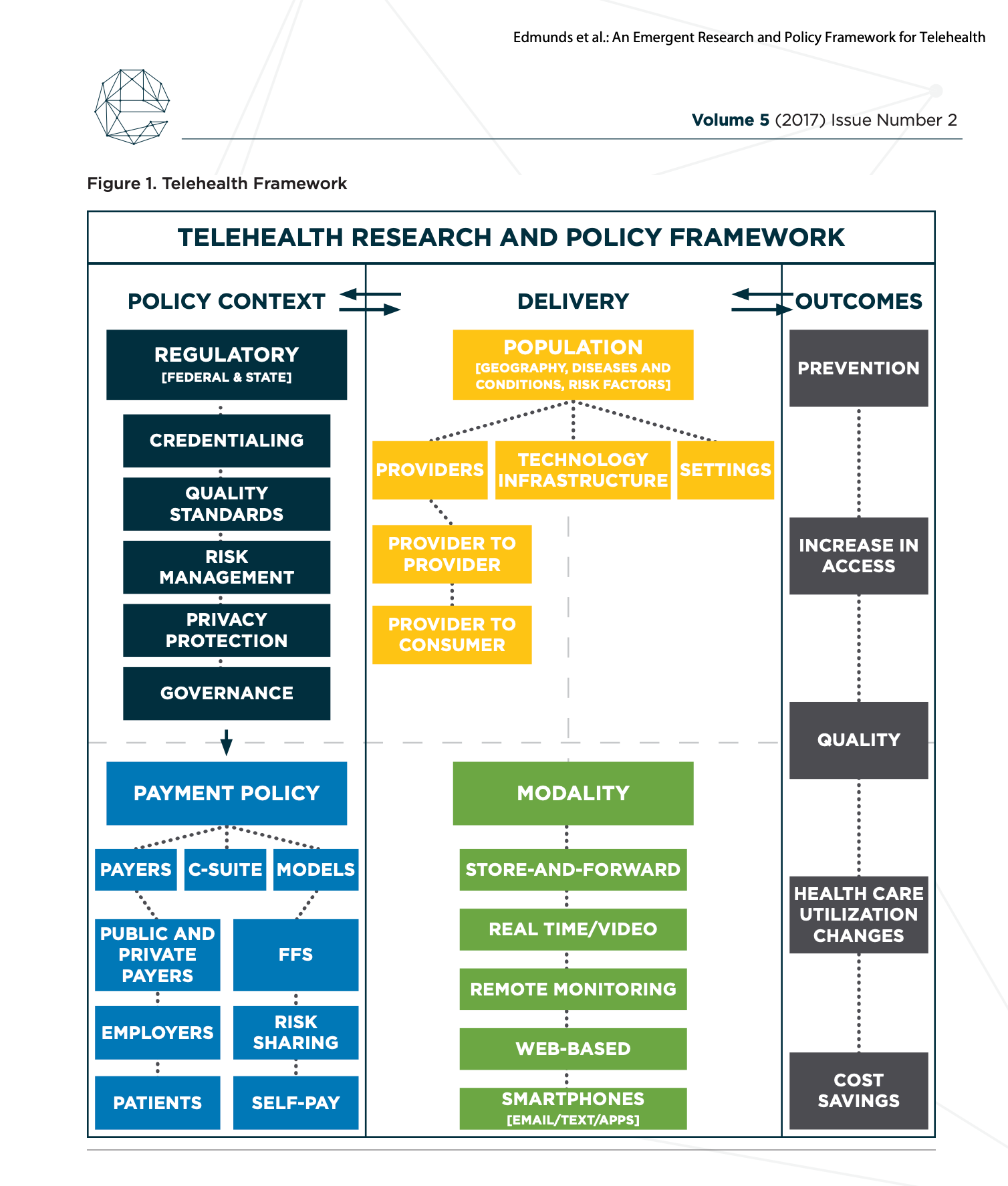

Supplement: S1 Fig — Republished from Edmunds et al. [13] under a CC BY license, with permission from the author, original copyright (2017). (TIFF) [file pone.0258839.s001.tiff]
