# Supplementary material for: Changes to telehealth practices in primary care in New Brunswick (Canada): A comparative study pre and during the COVID-19 pandemic
Source: PLoS One. 2021 Nov 23;16(11):e0258839. doi: 10.1371/journal.pone.0258839 (PMC8610241; doi:10.1371/journal.pone.0258839)
Supplement: S1 Questionnaire — (DOCX) [file pone.0258839.s002.docx]

**Access to primary healthcare in New Brunswick**

1. **In which region/community of New-Brunswick are you working as a general physician?**

___________________________________________________________________

1. **How many patients are currently on the physician case load?**
2. _______________ (number of patients)
3. I don’t know
4. Prefer not to answer
5. **Do you currently accept new patients?**
   1. Yes
   2. No
6. I don’t know
7. Prefer not to answer
8. **Which statement most resembles your way of scheduling appointments?**
   1. All appointments are made in advance, in the case of an emergency, the request for an appointment is either refused or added to an appointment that is already taken (double booking).
   2. A small portion (one third or less) of time slots are reserved for emergencies or same-day appointment requests
   3. The majority of the time slots (50% or more) are reserved for appointments the same day regardless of the type of appointment.
   4. No appointments are made; if a patient calls and no appointment is available, they call back the next day
   5. Other, please specify : _______________________________________________
9. I don’t know
10. Prefer not to answer
11. **Do you (the physician) of this practice also work in other settings (hospital, research, etc)?**
    1. Yes
    2. No
12. I don’t know
13. Prefer not to answer

**5.1 If yes, how many hours per week do you (the physician) spend in the family medicine practice?**

1. ________ (number of hours per week)
2. I don’t know
3. Prefer not to answer
4. **Does your practice describe itself as a family medicine team (a group of doctors working together, consisting of one or more family physicians, nurse practitioner)?**
5. Yes
6. No
7. I don’t know
8. Prefer not to answer
   1. **If yes, how many primary health care providers (family physicians, nurse practitioners) practice in your team?**
9. ______________ (number of physicians and nurse practitioners)
10. I don’t know
11. Prefer not to answer
12. **Are there any healthcare professionals other than family physicians or nurse practitioners who work in your practice?**
13. Yes
14. No
15. I don’t know
16. Refuse

**7.1 If yes, which ones?**

1. Occupational therapist
2. Psychologist
3. Social worker
4. Dietitian
5. Physiotherapist
6. Other, please specify: ___________________________________________
7. I don’t know
8. Prefer not to answer

**7.2 If yes, how many?**

1. ________ (number of professionals)
2. I don’t know
3. Prefer not to answer
4. **Which of the following statements applies most to your practice regarding the remuneration of the physician(s):**
5. Fee-for-service: the doctor is paid an amount fixed by the government for each service offered
6. Compensation per patient: the doctor is paid a fixed amount for each patient on his case load
7. Salaried: a fixed salary, regardless of the number of patients on the case load or the number of services offered
8. Mixed remuneration: a mix of the remuneration methods above
9. Other, please specify: ___________________________________________
10. I don’t know
11. Prefer not to answer
12. **Do you measure the quality of the patient experience?**
13. Yes
14. No
15. I don’t know
16. Prefer not to answer

**9.1 If yes, how?**

________________________________________________________________________________________________________________________________________________________________________________________________________________________

1. **Do you monitor preventive care in the office (for example, annual physical exams, education, prevention, etc)?**
2. Yes
3. No
4. I don’t know
5. Refuse
6. **Do you compare your performance with benchmark data or other practices in the province?**
7. Yes
8. No
9. I don’t know
10. Refuse
11. **Identify each technology you used regularly prior to the Covid-19 pandemic**
12. Emails
13. Electronic medical records
14. Online booking systems for patients
15. Text messages with patients (for appointment reminder, for example)
16. Voicemail
17. Telemedicine
18. Consultation by phone
19. Telework from home
20. Other, please specify: ___________________________________________
21. I don’t know
22. Prefer not to answer
23. **How many patients were seen per day (on average) before the Covid-19 pandemic?**
    1. __________ (number of patients)
24. I don’t know
25. Prefer not to answer
26. **How many patients are seen per day (on average) since the beginning of the Covid-19 pandemic?**
    1. _______ (number of patients)
27. I don’t know
28. Prefer not to answer
29. **If a client calls the practice today (during the pandemic) with a relatively urgent need, how many days, on average, will they wait for an appointment?**
    1. _____________ (number of days)
30. I don’t know
31. Prefer not to answer
32. **What tools (if any) have you added to your medical practice since the beginning of the Covid-19 pandemic?**
33. Email
34. Electronic medical charts
35. Online booking system
36. Texting
37. Voicemail
38. Telemedicine
39. Consultation by phone
40. Working from home
41. Nothing changed
42. Other, please specify: ___________________________________________
43. I don’t know
44. Prefer not to answer
45. **Do you offer regular office hours (~40 hours per week Monday to Friday) since the beginning of the Covid-19 pandemic?**
46. Yes, I am still working 40-hour weeks
47. No, I am working more than 40-hour weeks
48. No, I am working less than 40-hour weeks
49. I don’t know
50. Prefer not to answer
51. **Have you been offering after hours services since the Covid-19 pandemic?**
52. Yes
53. No
54. I don’t know
55. Prefer not to answer

**18.1 If yes, when are you offering after-hours services**

1. Evenings: ______ hours per week (0-40)
2. Weekends: ______ hours per week (0-40)
3. I don’t know
4. Prefer not to answer
5. **Have the hours per week where your office takes calls for appointments changed during the Covid-19 pandemic?**
6. No change
7. More hours since the pandemic
8. Less hours since the pandemic
9. I don’t know
10. Prefer not to answer

**19.1 At this time your office accepts calls:**

On Monday from ________ until ________h and _________ until __________ h

On Tuesday from ________ until ________h and _________ until __________ h

On Wednesday from ________ until ________h and _________ until ________ h

On Thursday from ________ until ________h and _________ until __________ h

On Friday from ________ until ________h and _________ until ____________ h

On Saturday from ________ until ________h and _________ until __________ h

On Sunday from ________ until ________h and _________ until __________ h

**19.2 Is that case load (number of patients) different since the Covid-19 pandemic?**

1. Yes, it is now higher since the pandemic
2. Yes, it is now lower since the pandemic
3. No, it has remained the same since the pandemic
4. I don’t know
5. Prefer not to answer
6. **Which changes in your practice should be maintained once the Covid-19 pandemic is over?**

________________________________________________________________________________________________________________________________________________________________________________________________________________________

**________________________________________________________________________**

1. **What are the challenges in your medical practice during the Covid-19 pandemic?**

________________________________________________________________________________________________________________________________________________________________________________________________________________________________________________________________________________________________

**Thank you for participating! Do you have any question or comments you think we should add to our research project?**

________________________________________________________________________________________________________________________________________________________________________________________________________________________________________________________________________________________________________________________________________________________________________
